# TIMs, TAMs, and PS- antibody targeting: implications for cancer immunotherapy

**DOI:** 10.1186/s12964-020-0521-5

**Published:** 2020-02-22

**Authors:** Adam S. Dayoub, Rolf A. Brekken

**Affiliations:** 1 Division of Surgical Oncology, Department of Surgery, Hamon Center for Therapeutic Oncology Research, University of Texas Southwestern Medical Center, 6000 Harry Hines Blvd., Dallas, TX 75390-8593 USA; 2 Department of Pharmacology, University of Texas Southwestern Medical Center, Dallas, TX USA

**Keywords:** Phosphatidylserine, Tumor, TAM, TIM, Antibody, Oncology, Immunotherapy, Tumor, Treatment, Clinical trial, Cancer

## Abstract

Immunotherapy for cancer is making impressive strides at improving survival of a subset of cancer patients. To increase the breadth of patients that benefit from immunotherapy, new strategies that combat the immunosuppressive microenvironment of tumors are needed. Phosphatidylserine (PS) signaling is exploited by tumors to enhance tumor immune evasion and thus strategies to inhibit PS-mediated immune suppression have potential to increase the efficacy of immunotherapy. PS is a membrane lipid that flips to the outer surface of the cell membrane during apoptosis and/or cell stress. Externalized PS can drive efferocytosis or engage PS receptors (PSRs) to promote local immune suppression. In the tumor microenvironment (TME) PS-mediated immune suppression is often termed apoptotic mimicry. Monoclonal antibodies (mAbs) targeting PS or PSRs have been developed and are in preclinical and clinical testing. The TIM (T-cell/transmembrane, immunoglobulin, and mucin) and TAM (Tyro3, AXL, and MerTK) family of receptors are PSRs that have been shown to drive PS-mediated immune suppression in tumors. This review will highlight the development of mAbs targeting PS, TIM-3 and the TAM receptors.

Video Abstract

Video Abstract

## Background

Michele Peyrone in 1845 described a molecule that had anti-cancer activity called “Peyrone salt,” Alfred Werner in 1893 deduced the structure of the salt, and Barnett Rosenberg in 1965 discovered the biological effects of this salt, a substance that the field of oncology now knows as cisplatin [1, 2]. Since 1965, life-changing advancements in chemotherapy design and utilization have been made but hurdles for the systemic treatment of cancer remain. The realization that the immune system can be harnessed to fight a patients’ own disease has provided a new arsenal of strategies for cancer therapy [3–11]. Immunotherapy is now first line therapy for some cancers [12–15] and the immunotherapy options have grown substantially, to include vaccines, immune checkpoint blockade, immune agonists and chimeric antigen receptor (CAR) T-cell therapy [16–19]. To expand the impact of immunotherapy, signaling pathways that drive tumor evasion of immune surveillance are under robust investigation. Phosphatidylserine (PS), an anionic phospholipid present in all mammalian cells has been studied for the past two decades as a critical immunosuppressive feature that tumors use to mask their presence from the immune system. Research has shown targeting PS or PS-receptors (PSR) with monoclonal antibodies (mAb) can alter PS-mediated immunosuppression and facilitate the induction of an innate and adaptive anti-tumor immune response. This review will cover the current literature of targeting PS and PSRs by monoclonal antibodies for the treatment of cancer.

### Phosphatidylserine

Lipid bilayers envelop eukaryotic cells and organelles to subdivide the cell into distinct working compartments. Phospholipid bilayers account for almost three-quarters of mammalian cell content. The major phospholipids in the cell include phosphatidylcholine (PC) and phosphatidylethanolamine (PE), which make up 45–50% and 30–40% of the phospholipids in cell, respectively. Other phospholipids, which are less abundant but integral to membrane function and homeostasis are phosphatidylinositol (PI), PS, and phosphatidic acid (PA) [20, 21]. While PS is a minor constituent in eukaryotic cells, PS-induced processes are highly conserved and have significant physiological functions.

PS is asymmetrically distributed to the inner leaflet of the plasma membrane in a highly conserved ATP-dependent process [22, 23]. PS is redistributed or flipped to the outer leaflet of the plasma membrane during or as result of certain cellular contexts or processes, the most well-described of which is apoptosis [24]. PS redistribution during apoptosis is facilitated by floppases and scamblases [24, 25]. TMEM16F is a Ca^2+^-dependent membrane associated phospholipid scramblase that can translocate PS to the outer leaflet of the plasma membrane [24]. However, TMEM16F is not required for exposure of PS in apoptotic cells. XKR8 is a caspase 3/7-activated phospholipid scramblase that appears to be responsible for PS exposure as a consequence of apoptosis [24]. Other scramblases, members of the TMEM16 and XKR families also exist and may function in a tissue selective manner and/or function as alternative scramblases that translocate PS [24]. Apoptosis induction and membrane phospholipid asymmetry collapse can be caused by perturbations in ion (Ca^2+^, K^+^, Na^+^) channels, the production of reactive oxygen species (ROS) via cell stress or mitochondrial-initiated apoptosis and caspase activation via DNA damage, radiation damage, and metal toxicity [26]. After PS is redistributed to the cell surface it can function as an “eat me” signal that initiates efferocytosis [27]. Aside from externalization on apoptotic bodies, PS has also been reported to be externalized on other cell types such as immune cells and cancer cells. For example, PS is found on myeloid-derived suppressor cells (MDSCs), monocytes, macrophages, active B cells, dendritic cells (DCs) activated mast cells and T cells [28–34]. In the tumor microenvironment (TME), exposed PS can also be found on tumor cells, secreted microvesicles and tumor endothelial cells [35]. PS-mediated efferocytosis initiates a highly conserved process that prevents local and systemic immune activation via signaling by PSRs. Importantly, PSR activation on immune cells creates an immunosuppressive milieu that tumor cells use as immune-camouflage [28]. Immune cells including MDSCs, CD4^+^ and CD8^+^ T cells, DCs, macrophages, B cells, and natural killer cells (NKs) express PSRs [36, 37].

PSRs are separated into two distinct families: those that bind PS directly and those that bind PS via a bridging protein (see Table 1). PSRs that are direct PS binders are exemplified by the T cell/transmembrane, immunoglobulin, and mucin (TIM) family of receptors, which are well characterized for their immune regulatory activity driven by PS binding [38, 39]. PSRs that are indirect PS binders are exemplified by the Tyro3, AXL, and MerTK (TAM) receptor tyrosine kinase (RTK) family that use gamma carboxylated growth arrest-specific 6 (Gas6) and Protein S (ProS) as the bridging molecule linking the receptor to PS [40]. TAM receptors have also been characterized for immune regulatory activity after PS-induced activation [15, 36, 40]. Given that PS-mediated signaling can induce local immune suppression and that tumors exploit this evolutionarily conserved pathways to evade immune detection, it is reasonable to suggest that interfering with PSR activity could augment anti-cancer immune therapy. Multiple strategies to interfere with PSR activity have been developed including monoclonal antibodies (mAbs) that target PS [41, 42].

**Table 1:** PS-receptor (PSR) binding to PS via direct or indirect binding

PSR	Direct or indirect PS-binding	Bridging ligand
TIM-1,3, and 4	Direct	
TAMs	Indirect	Gas-6, ProS
Stabilin 1, and 2	Direct	
RAGE	Direct	
CD300a	Direct	
BAI1	Direct	
Αvβ3–5	Indirect	MFG-E8

## Background and current developments with mAb immunotherapy targeting

### TIM-3

In humans there are three genes that make up the TIM family: TIM-1, − 3, and − 4 [43]. TIM genes encode type 1 membrane spanning proteins and TIM receptors consist of four well-defined regions: the variable immunoglobulin domain (IgV), mucin domain, transmembrane region, and intracellular stem [44]. All 3 TIM receptors have been implicated as PSRs [45, 46]; however, inhibitory TIM-3 mAbs are further advanced and will be discussed here. It should be noted that no current TIM-1 or TIM-4 mAb clinical trials are ongoing although antibody-drug conjugates (ADCs) targeting these receptors are being developed [47]. TIM-3 is expressed in multiple types of cancers including sarcoma, cervical and gastric cancer, myeloma, melanoma, and lung cancer [43, 48–51] and expression of TIM-3 correlates with worse outcome [43, 44, 46]. TIM-3 is also expressed on different immune cell types. For example, TIM-3 has been reported on DC populations, which suggests that antigen presentation and phagocytosis can be affected by this PSR [52]. TIM-3 expression is also found on CD8^+^ T cells, regulatory T cells (Tregs), and NK cells [50]. Furthermore, M2-like macrophages show higher levels of TIM-3 expression than M1-like macrophages [44, 53]. Consistent with human expression data, TIM-3 expression on peripheral blood monocytes and tumor-associated macrophages has been shown to correlate with disease progression in a murine model of hepatocellular carcinoma [44, 54]. Interestingly, PS is the only nonprotein known to bind to the family of TIM receptors. It should be noted aside from PS, TIM-3 has been identified to interact with several other proteins implicated in immune regulation, including galectin-9 (gal-9), carcinoembryonic antigen cell adhesion molecule 1 (CEACAM-1), and high-mobility group protein box 1 (HMGB-1) [14, 55–59]. PS binding directly to TIM-3 has been confirmed and it has been shown to induce efferocytosis in phagocytic cells [60] although the affinity of TIM-3 for PS is weaker than TIM-1 and 4 [61] [62]. It has been proposed that PS and TIM-3 interactions promote immune cell exhaustion since PS is involved in immune cell tolerance. Silva et al. working on reversing NK cell exhaustion hypothesized that since PS is on the surface on apoptotic bodies, it might stimulate NK cell exhaustion after effector-induced tumor cell death [63]. In addition, TIM-3^+^ APCs phagocytize apoptotic bodies but T-cells that express TIM-3 form conjugates that are not capable of phagocytosis. However, Freeman et al. proposes that cross-linking conjugates on T-cells by apoptotic bodies may provide an immunostimulatory signal to T-cells [45]. This effect would be induced because of the binding of TIM-3 on Th1 or Th17 cells via galectin-9 [64, 65].

Immune cells that express TIM-3 promote immune tolerance to tumors and thus therapeutic mAbs that target TIM-3 have been developed and tested preclinically and clinically. Studies in multiple animal models have shown that antibody-mediated Tim-3 inhibition enhances the activity of immune checkpoint blockade [66–68] although detailed analysis of the tumor immune landscape is still incomplete after Tim-3 inhibition. High levels of TIM-3 correlate with exhausted CD8^+^ T cells in melanoma patients and anti–TIM-3 mAb treatment reversed this phenotype [69]. Non–small-cell lung cancer (NSCLC) patients were found to have high expression of TIM-3 on CD4^+^ and CD8^+^ T cells [69]. TIM-3 has also been found on tumor-infiltrating lymphocytes (TILs) in head and neck cancer, renal cell carcinoma, gastric cancer, non-Hodgkin’s lymphoma, cervical cancer, prostate cancer, colorectal cancer, and hepatocellular cancer [70]. Furthermore, TIM-3 expression is now recognized as a marker of T cell exhaustion. This is illustrated by a recent study, where TIM-3^+^ TILs co-expressed programmed cell death protein 1 (PD-1) and lacked interleukin-12 (IL-12), tumor necrosis factor (TNF), and interferon gamma (IFNy) expression [68, 71]. This has ignited speculation that combining anti-TIM-3 with anti–PD-1 therapy might be a viable option to overcome T-cell exhaustion in patients and promote responses to immune checkpoint blockade. Furthermore, TIM-3 inhibition has been implicated as a possible strategy for priming response to other therapies such as Toll-like receptors (TLR) agonists to promote an active anti-tumor immune response. For example, blocking TIM-3 followed by TLR agonist treatment resulted in the expression of IL-12, interleukin-10 (IL-10), and interleukin-6 (IL-6) in hepatitis C monocytes, and this strategy may be applicable to cancer [72].

Antibodies against TIM-3 are being investigated in multiple clinical trials (see Table 2). NCT03680508 is a phase II trial, testing anti–TIM-3 mAb TSR-022 in combination with anti–PD-1 mAb TSR-042 in patients with hepatocellular carcinoma [73]. Early data suggests that blocking TIM-3 enhances cytotoxic T-cell–mediated tumor lysis [74, 75]. NCT02608268 is studying the effect of anti-TIM-3 in advanced malignancies. This phase I/II trial is evaluating anti–TIM-3 mAb as a single agent and in combination with PDR001 (anti–PD-1 antibody).

**Table 2:** Current clinical trials testing TIM-3–specific antibodies in cancer patients

Intervention (mAb)	Primary target	Study	Conditions	Clinical trial	Identifier
Sym023	TIM-3	Advanced solid tumor malignancies or lymphomas	Metastatic cancer, solid tumor, lymphoma	Phase 1 – recruiting	NCT03489343
TSR-022 TSR-042	TIM-3 PD-1	Advanced solid tumors	Advanced or metastatic solid tumors	Phase 1 –recruiting	NCT02817633
TSR-022 TSR-042	TIM-3 PD-1	Advanced liver cancer	Liver cancer	Phase 1 – not recruiting yet	NCT03680508
R07121661	TIM-3 and PD-1 (bispecific targeting)	Dose escalation study with advanced or metastatic solid tumors	Solid tumors, metastatic melanoma, NSCLC, SCLC	Phase 1 – recruiting	NCT03708328
MBG453	TIM-3	Recurring glioblastoma patients	Glioblastoma	Phase 1 – not recruiting yet	NCT03961971
MBG453 PDR001	TIM-3 PD-1	Single agent vs. combo study against advanced malignancies	Malignancies	Phase 1 – recruiting	NCT02608268
LY3321367 LY3300054	TIM-3 PD-1	Advanced relapsing/refractory solid tumors	Solid tumors	Phase 1 – recruiting	NCT03099109
LY3415244	TIM-3 and PD-1 (bispecific targeting)	Advanced solid tumors	Solid tumors	Phase 1 – recruiting	NCT03752177
BGB-A425 Tislelozumab	TIM-3 PD-1	Combo in advanced solid tumors	Local advanced solid tumors, metastatic solid tumors	Phase 1 –recruiting	NCT03744468

*Abbreviations*: *SCLC* small-cell lung cancer, *NSCLC* non–small-cell lung cancer

### TAM receptors

TAM receptors contribute to cancer development, growth and metastasis. The two most characterized TAM ligands are vitamin K-dependent proteins, Gas6 and ProS [76]. Gas6 and ProS bind PS via gamma carboxylation motif and are produced by multiple cell types, including tumor cells, immune cells and fibroblasts in the TME [77, 78]. TAM receptors expressed by phagocytic cells participate in efferocytosis and can induce a tolerogenic immune cell phenotype [79–81], thereby promoting tumor immune evasion. For example, TAM receptors have been found on macrophages, DCs, NK cells, T cells, and can indirectly affect T-cell functions in the TME [81]. Axl and MerTK are expressed in bone marrow-derived DCs and Gas6 has been shown to mediate reduced TLR response as measured by production of IL-6, tumor necrosis factor alpha (TNFα), and type I interferon after TLR agonist stimulation [81, 82]. In addition, Axl activation on macrophages and DC can result in the upregulation of negative TLR and cytokine regulators, suppressor of cytokine signaling-1 (SOCS1) and suppressor of cytokine signaling-3 (SOCS3), which further dampen immune activation [83]. Mouse models have shown that a lack of expression of TAM receptors or inhibition of TAM signaling can increase immune-mediated rejection of tumor cells [84, 85]. Additionally, TAM receptors prevent the induction of immune responses by preventing the activation of antigen-presenting cells (APCs) via PS binding with Gas6 or ProS [86]. TAM receptors, Axl and MerTK, are also expressed by tumor cells in many tumor types [81]. Activation of Axl/MerTK on tumor cells results in induction and maintenance of a mesenchymal-like tumor cell phenotype.

As a result, TAM receptors can drive epithelial plasticity or epithelial to mesenchymal transition (EMT) [40]. EMT is linked to tumor cell survival, therapy resistance, metastasis and immune suppression in multiple tumor types [87, 88]. Multiple strategies to inhibit TAM receptors have been developed. These include neutralizing mAbs, ADCs and small molecule inhibitors. Recent reviews on the validation of Axl and MerTK as therapeutic targets are available (78, Parinot, 2016 #145). Here we will provide an overview of mAbs targeting TAM receptors and how these agents impact the tumor microenvironment.

Pre-clinical studies with mAb targeting the TAM receptors have contributed to our understanding of the function of TAM receptors in cancer. Antibodies discussed in this section are shown in Table 3. Demarest et al. [89] published a robust study on a series mAbs specific for Tyro3 in melanoma cell lines. They identified mAbs that show moderate to high affinity to the extracellular domain of Tyro3 and a range of activity in blocking Gas6 binding to the receptor and inhibition of ligand-induced Tyro3 signaling. Chien et al. [90] engineered a human anti-Tyro3 mAb, Tyro3-hIgG, and reported that the mAb inhibited cell migration and invasion in human colon cancer cells and NIH3T3 fibroblasts. They also provided evidence that inhibition of Tyro3 can reverse EMT and enhance sensitivity of cancer cells to chemotherapy. These findings along with multiple other studies [91–96] have highlighted the contribution of Tyro3 to the tumor microenvironment. To our knowledge, Tyro3 specific mAbs have not advanced to clinical testing to date.

**Table 3:** TAM-targeting monoclonal antibodies

mAb	Species	Target
hTryo-3-ECD	Human	Tyro3
hTyro3-Ig	Human	Tyro3
hTyro3-ECD-Fc	Human	Tyro3
DAXL-88	Human and Mouse	Axl
BA3011	Human	Axl
YW327.6S2	Human	Axl
20G7-D9	Human	Axl
RGX-019	Human	MerTK
Murine-RGX-019	Mouse	MerTK
Mer590	Human	Mer

In contrast to Tyro3, numerous groups have developed mAbs specific for Axl. Multiple preclinical studies with the Axl mAb DAXL-88 have shown that it can inhibit tumor cell migration and invasion in vitro [97]. In addition, DAXL-88, which binds mouse and human Axl has shown impressive anti-tumor efficacy in mice bearing MDA-MB-231 xenografts [97]. BA3011 is another Axl targeting mAb that selectively binds to human Axl [98]. BA3011 showed efficacy in lung, prostate and pancreatic cancer xenograft models [98] and has been developed as an ADC, CAB-AXL-ADC with a proprietary protein as the drug. CAB-AXL-ADC has entered clinical testing (trial identifier NCT0342527). Other therapeutic anti-Axl mAbs that have shown efficacy in preclinical models of cancer include YW327.6S2 (YW) and 20G7-D9. YW is a phage-derived mAb that showed anti-tumor efficacy in preclinical models of NSCLC and breast cancer models [99]. YW recognizes mouse and human Axl [99], inhibits the binding of Gas6 to Axl in a dose-dependent–mediated manner and downregulates Axl receptor expression. In xenograft studies, YW reduced vascular density and inhibited inflammatory cytokine expression from tumor-associated macrophages [99]. YW also enhanced the efficacy of EGFR inhibition with erlotinib in NSCLC xenografts [100] and reduced metastasis [99]. Clinical studies with YW are likely and could include combination with anti-vascular endothelial growth factor (VEGF) strategies. 20G7-D9, has also been evaluated in multiple breast cancer models, including xenograft and patient-derived xenografts [101]. 20G7-D9 inhibited tumor growth and bone metastasis lesions in a tumor cell Axl-dependent manner, highlighting the importance of tumor cell Axl expression to tumor progression and the efficacy of Axl targeted agents [101]. In addition, 20G7-D9 induced Axl degradation and inhibited Gas6-dependent cell signaling, cell migration and EMT [101]. 20G7-D9 is being developed as a therapeutic mAb and an ADC. Axl mAbs are currently being evaluated in preclinical studies in combination with immune therapy in multiple indications.

Antibodies specific for MerTK have also been developed and tested in preclinical cancer models. RGX-019 is a MerTK targeting mAb that prevents Gas6 induced phosphorylation of AKT resulting in inhibition of melanoma cell growth and colony formation [102]. In addition, the same study showed RGX-019 prevented MDA-MB-231 breast tumor growth in vivo. Cummings et al. [103] reported on another MerTK targeting mAb, Mer590 that reduced MerTK levels in NSCLC cell lines in vitro. Mer590 inhibited STAT6, AKT and ERK1/2 activation and resulted in MerTK down-regulation, resulting in increased apoptosis and decreased colony formation.

At the time of writing this review there are no active clinical trials involving mAbs targeting TAM receptors; however, it is anticipated that multiple TAM mAbs will enter clinical testing soon. Preclinical studies with small molecular weight inhibitors of MerTK and Axl have been shown to alter the tumor immune landscape to favor anti-tumor immune activity [77, 104, 105], thus it is anticipated that antibody-mediated inhibition of TAM receptors will also alter the tumor immune landscape. TAM receptors have a clear function in immunosuppressive signaling in cancer and it is likely that mAbs targeting TAMs will be evaluated in the context of immune checkpoint blockade in cancer patients.

### Phosphatidylserine

Antibodies that target PS were developed by Philip Thorpe’s laboratory to specifically home to tumor vasculature (reviewed in Belzile [27]). The realization that PS is externalized on tumor endothelial cells but not on endothelial cells in normal tissues was the result of studies on the efficacy of a coagulation-inducing vascular targeting agent (VTA) specific for vascular cell adhesion molecule 1 (VCAM1) in tumor-bearing mice [106]. Ran et al. [106] showed that a VCAM1-targeted VTA localized to tumor blood vessels and vessels in some normal organs, including cardiac blood vessels; yet coagulation was only induced in the tumor. They went on to demonstrate that the selective efficacy was due to exposure of PS on the luminal surface of tumor endothelial cells, which supported initiation of the coagulation cascade. This led to the development of a series of mAbs that target PS. Unlike other PS binding agents, including annexin V, the mAbs bind PS in a calcium-independent manner [106–108]. Robust in vivo localization studies in mice revealed that PS-targeting mAbs and annexin V specifically localize to tumor vasculature but were not present normal organs evaluated [27, 106–108]. These observations suggested that targeting anionic lipids, such as PS, was viable and potentially useful as an anti-cancer strategy.

**Fig. 1:**
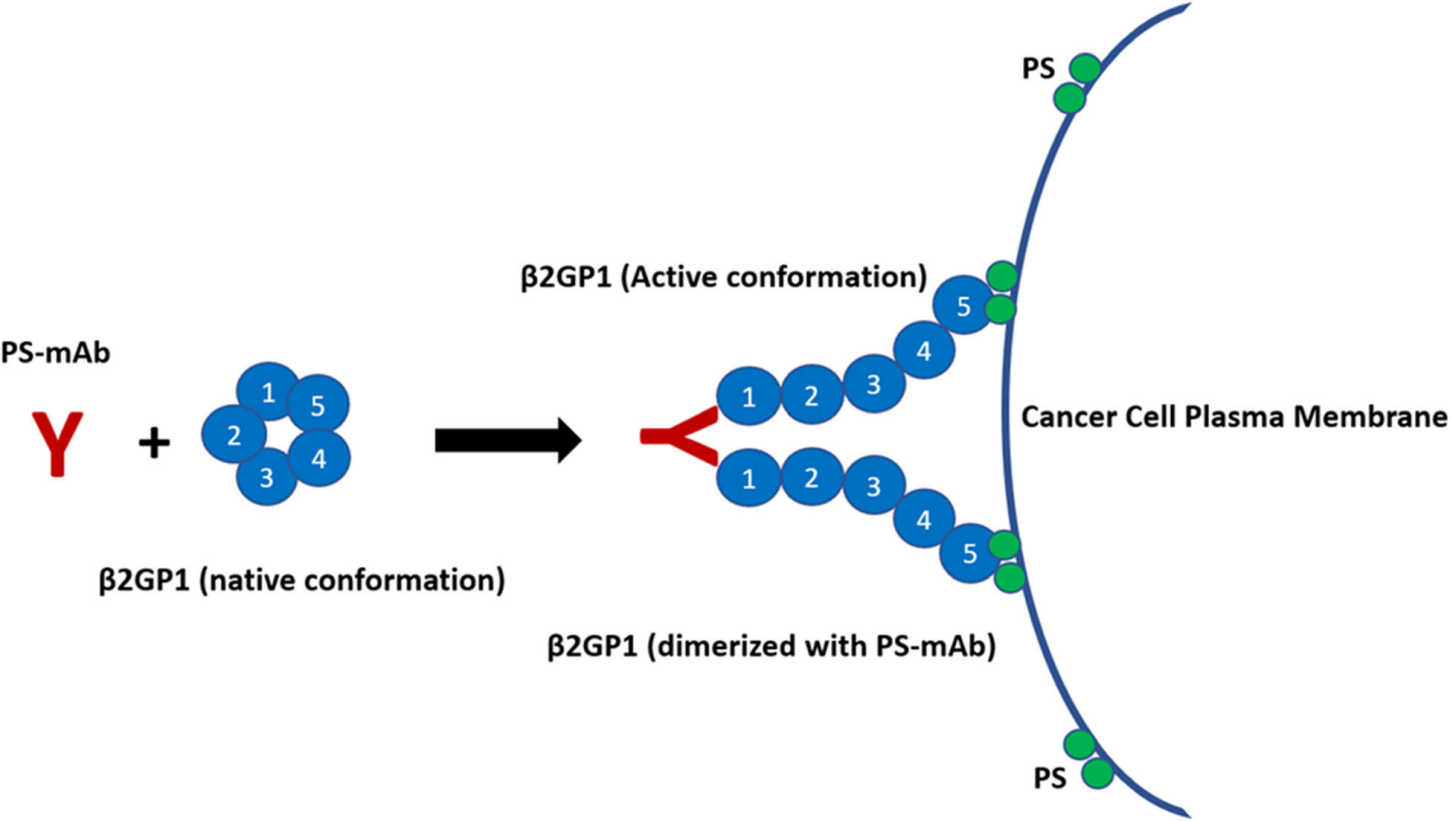
Diagram depicting PS-targeting mAb and β2GP1 binding to PS on a cell surface. Proposed mechanism of PS-targeting mAb binding to exposed PS in a β2GP1-dependent manner. Not drawn to scale

The majority of PS-targeting mAbs developed by the Thorpe laboratory bind PS via a serum cofactor, β2 glycoprotein 1 (β2GP1) (see Table 4). β2GP1, a known PS interacting protein [109, 110], is a 5-domain protein found in abundance in sera (~ 200 μg/mL). In its native state, β2GP1 is in a circular protein conformation [111]. Studies indicate β2GP1 adopts an open “J-shape” structure in the presence of select antibodies and other activating proteins/lipids [110]. PS-targeting mAbs, including 3G4 and its derivatives, bind and dimerize β2GP1 such that domain 5 of each β2GP1 in the complex binds with high affinity to PS on the plasma membrane [27]. Figure 1 presents a schematic depicting the interaction of PS-targeting mAbs with β2GP1 and PS. The PS-targeting mAbs while initially developed to selectively bind to tumor vasculature were found to have anti-tumor efficacy in preclinical tumor models [112]. In fact, the mAbs have been shown to enhance the efficacy of standard chemotherapy [58, 113, 114] and radiation therapy [115, 116] in multiple mouse cancer models. β2GP1 is the primary antigen associated with anti-phospholipid syndrome, an autoimmune disorder characterized by the production of anti-phospholipid antibodies that enhance thrombosis and complications during pregnancy and is associated with systemic lupus erythematosus. Mineo et al. found that one of the Thorpe PS-targeting mAbs (1 N11) prevents the pathogenesis induced by anti-phospholipid antibodies in preclinical models [117]. These data suggest that not all antibodies that bind β2GP1 are the same and also highlight that 1 N11 or other therapeutic anti-PS targeting mAbs might have utility for the treatment of APS.

**Table 4:** PS-Targeting monoclonal antibodies (mAb)

mAb	Species	Isotype
3G4	Mouse	IgG3
2aG4	Mouse	IgG2a
Bavituximab	Human chimeric 3G4	IgG1
1 N11	Fully human	IgG1
Mch1 N11	Mouse chimeric 1 N11	IgG2a

Investigation of the anti-cancer mechanism(s) of action of PS-targeting mAbs showed that 40% of blood vessels were bound by the mAb in orthotopic human breast xenografts [112]. Additionally, the mAb appeared to induce antibody dependent cellular cytotoxicity (ADCC) directed towards endothelial cells and this effect was magnified in the presence of chemotherapy [113]. These results suggest that chemotherapy induces increased PS externalization and that PS-targeting mAbs alter immune cell phenotype as macrophages in the TME typically are not capable of performing ADCC. Evidence supporting that PS-targeting can alter immune cell phenotype includes the observation that tumor vasculature was reduced after treating with a PS-targeting mAb + docetaxel and this corresponded to a 4 and 14-fold increase in macrophage infiltration into tumors treated with a PS-targeting mAb alone or in combination with docetaxel, respectively [113]. PS-targeting also enhanced the efficacy of PRIMA-1 (APR-246), a therapeutic agent that reactivates mutant p53 [118, 119]. In addition, PS-targeting showed similar anti-tumor efficacy when combined with an onco-adenovirus, Delta-24-RGD, that replicates in tumors and promotes high PS exposure after viral infection [120]. Supporting these observations, additional studies provided evidence that PS-targeting alters myeloid cell phenotype in human tumor xenografts. Yin et al. [121] found that PS-targeting mAbs dramatically shifted the phenotype of macrophages from an M2-like to a M1-like phenotype and that the mAb induced the differentiation of MDSCs to M1-like macrophages and mature DCs and reduced the expansion of immunosuppressive cell types, including MDSCs and Tregs in the TME [121]. Additionally, the authors demonstrated through electron microscopy that the PS-targeting mAbs interact with immune cells through extracellular vesicles and also provided evidence that this immune reprogramming is dependent on the Fc portion of the PS-targeting mAb suggesting that the change in immune cell phenotype is dependent upon a) blocking PS-PSR interaction and b) Fc receptor engagement on the immune cell. One of the key consequences of PS-targeting mAb activity is DC maturation, which can presumably impact induction of an adaptive immune response.

**Fig. 2:**
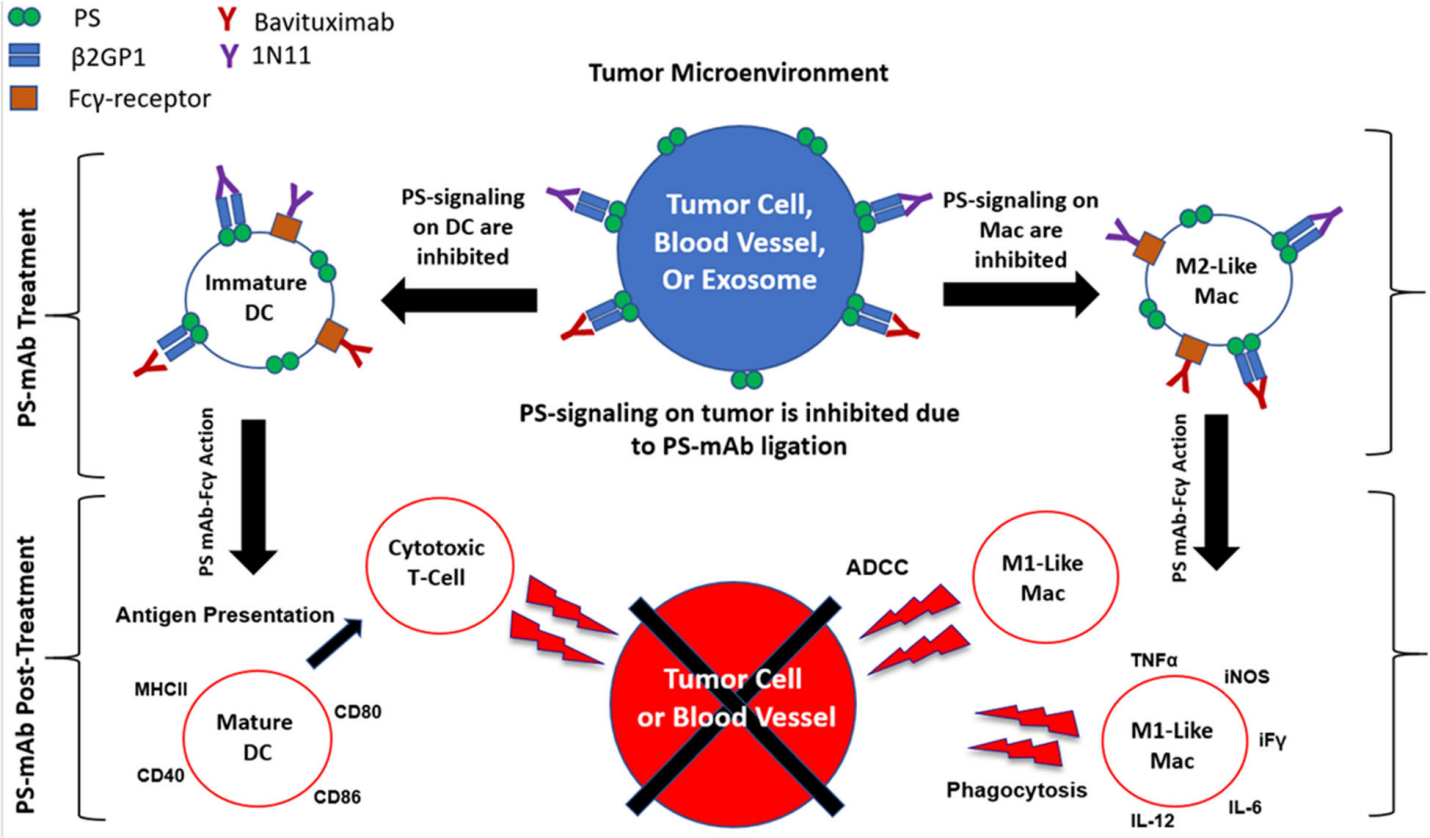
Diagram of multiple immune activation cascades upon treatment with PS-targeting mAb. Proposed pathways altered by PS-targeting mAbs that could result in improved anti-tumor immune activity

The first evidence that PS-targeting mAbs could facilitate an adaptive immune response was shown by He et al [115]. The authors found that radiation in combination with a PS-targeting mAb induced long-term survival in rats bearing orthotopic syngenic F98 glioma cells. Additionally, splenocytes from long-term survivors showed cytotoxic activity against F98 tumor cells in vitro [115]. Furthermore, combination of PS-targeting mAbs with immune checkpoint blockade (anti- cytotoxic T-lymphocyte-associated protein 4 (CTLA-4) or anti-PD-1) has now been evaluated in breast and melanoma syngenic models of cancer in immunocompetent mice [122, 123]. For example, Freimark et al. showed that PS-targeting enhanced the efficacy of anti-PD-1 and altered the immune landscape of tumors by increasing T-cell infiltration, proliferation and activation [123]. Taken together these data strongly suggest that the anti-cancer efficacy of PS-targeting mAb results from targeting tumor vasculature and altering the immune microenvironment of tumors by interfering with PS-mediated immune suppression (Fig. 2).

Bavituximab, a chimeric PS-targeting mAb has been evaluated in multiple clinical trials where it was found safe and well tolerated [124, 125]. Given that β2GP1 has been implicated in regulating coagulation [126] the effect of bavituximab on coagulation was evaluated closely. The phase I study saw a modest prolongation of activated partial thomboplastin timed in vitro at the highest doses given but a maximum tolerated dose of bavituximab was not identified [124]. Given the potential immune modulatory activity of bavituximab it was studied using 3D ex vivo-cultured tumor spheroids from NSCLC patients who had low PD-1 levels. Bavituximab incubation with the spheroids resulted in the increase of multiple immune-activating cytokines such as Granulocyte-Macrophage Colony Stimulating Factor (GM-CSF), IFNγ, and TNF-α. Similar results were found in patient 3D spheroids that had low levels of PD-L1 [127]. Furthermore, Secondary analysis of a Phase III trial (SUNRISE, NCT01999673) evaluating docetaxel alone vs. docetaxel and bavituximab as a second line therapy in non-small cell lung cancer (NSCLC) showed that adding PD-1 inhibition following progression was more efficacious in patients that were treated with bavituximab + docetaxel than patients treated with docetaxel alone. Additionally, analysis of circulating cytokines demonstrated that low pretreatment serum levels of IFNγ was associated with increased efficacy with the combination bavituximab and immunotherapy [128, 129]. This suggests that PS-targeting mAbs may increase the priming T cells and highlights that the combination of PS-targeting mAbs + immune checkpoint blockade should be studied further. Ongoing trials testing bavituximab are listed in Table 5.

**Table 5:** Current clinical trials testing PS-specific antibodies in cancer patients

Intervention (mAb)	Primary target	Study	Conditions	Clinical trial	Identifier
Bavituximab Temozolomide Radiation	PS DNA (alkylating agent) DNA (damaging energy)	Bavituximab with radiation in diagnosed glioblastoma	Glioblastoma	Phase 2 – not recruiting	NCT03139916
Bavituximab Pembrolizumab	PS PD-1	Patients with advanced hepatocellular carcinoma	Hepatocellular cancer	Phase 2 - recruiting	NCT03519997
Bavituximab Pembrolizumab	PS PD-1	Advanced Gastric Cancer after one prior standard therapy regimen	Gastric Cancer Gastro-Esophageal Cancer	Phase 2 - recruiting	NCT04099641

## Conclusions

PS is an important modulator of the tumor immune microenvironment. PS-mediated immune suppression is an evolutionarily conserved pathway that tumors hijack to avoid immune surveillance. This is driven by PS interacting with PSRs, which are expressed on immune cells, endothelial cells and tumor cells. Inhibition of PSR signaling by direct targeting of PSRs or by targeting PS is currently being investigated in preclinical and clinical trials. This mini-review highlighted the contribution of TIM and TAM receptors to PS-mediated signaling in the TME; however, there are additional PSRs that induce efferocytosis and might also contribute to immune suppression. These PSRs including BAI1, CD300e, Stabilin-1 and others are worth considering in the context of anti-cancer immune therapy. Additionally, canonical signaling induced by PS is only beginning to be defined.

For instance, it is not clear if PSRs fall into classes of receptors based on signaling or cell type or potency for induction of efferocytosis and local immune suppression. We also provided an overview of the effect of PS-targeting mAbs in altering the immune landscape of tumors. While PS-targeting has advanced to clinical testing in multiple indications there are several unanswered questions that remain. The biochemical mechanism of action of the PS-targeting mAbs is yet to be fully delineated. Further it is not clear if PS-targeting mAbs interfere with all PSR signaling or a subset of PSRs. Additionally, the effect of PS-targeting mAbs on tumor cell phenotype is unexplored. This seems a potentially fruitful area of investigation given the importance of PSRs in the progression of multiple tumor types.

Understanding which patients might benefit from targeting the PS-PSR pathway is a focus for multiple groups. However, this is a challenging task given the complexity of PS-PSR biology in the TME. Highlighted by the fact that there are multiple potential sources of PS and over a dozen PSRs that might participate in PS-mediated signaling on many cell types. Thus, further research on PSRs in the context tumor immune suppression is certainly warranted.

## Data Availability

Not applicable.

## Abbreviations

ADC: Antibody-drug conjugate
ADCC: Antibody-dependent cellular cytotoxicity
CAR: Chimeric antigen receptor
CEACAM-1: Carcinoembryonic antigen cell adhesion molecule 1
CTLA-4: Cytotoxic T-lymphocyte-associated protein 4
DC: Dendritic cell
EMT: Epithelial to mesenchymal transition
Gal-9: Galectin-9
Gas6: Growth arrest-specific 6
GM-CSF: Granulocyte-Macrophage Colony Stimulating Factor
HMGB-1: High-mobility group protein box 1
IgV: Immunoglobulin domain V
IL-10: Interleukin-10
IL-12: Interleukin-12
IL-6: Interleukin-6
INFγ: Interferon gamma
mAb: Monoclonal antibodies
MDSC: Myeloid-derived suppressor cells
NK: Natural killer cell
NSCLC: Non–small-cell lung cancer
PA: Phosphatidic acid
PC: Phosphatidylcholine
PE: Phosphatidylethanolamine
PI: Phosphatidylinositol
ProS: Protein S
PS: Phosphatidylserine
PSR: Phosphatidylserine receptor
ROS: Reactive oxygen species
RTK: Receptor tyrosine kinase
SOCS1: Suppressor of cytokine signaling-1
SOCS3: Suppressor of cytokine signaling-3
TAM: Tyro3, AXL, and MerTK
TIL: Tumor-infiltrating lymphocytes
TIM: T-cell/transmembrane, immunoglobulin and mucin
TLR: Toll-like receptor
TME: Tumor microenvironment
TNF: Tumor necrosis factor
TNFα: Tumor necrosis factor alpha
Treg: Regulatory T cells
VCAM1: Vascular cell adhesion molecule 1
VEGF: Anti-vascular endothelial growth factor
VTA: Vascular targeting agent
YW: YW327.6S2
β2GP1: β2 glycoprotein 1
